# The effects of zinc supplementation on olfactory disorders: a systematic review with meta-analysis

**DOI:** 10.1016/j.bjorl.2026.101914

**Published:** 2026-10-28

**Authors:** Marco Aurélio Fornazieri, Heloísa Bortholazzi Fornazieri, Nayara Anitelli Artero, Ellen Cristine Duarte Garcia, Fábio de Rezende Pinna, Richard Louis Voegels

**Affiliations:** a Universidade Estadual de Londrina, Department of Clinical Surgery, Londrina, PR, Brazil; b Pontifícia Universidade Católica do Paraná, Department of Medicine, Londrina, PR, Brazil; c Universidade de São Paulo, Department of Otorhinolaryngology, São Paulo, SP, Brazil

**Keywords:** Zinc, Smell, Olfaction disorders, Anosmia

## Abstract

**Objectives:**

This systematic review aimed to evaluate the efficacy of zinc supplementation for treating olfactory disorders, including quantitative and qualitative symptoms, in adults.

**Methods:**

We conducted a comprehensive search of the Cochrane Central Register of Controlled Trials, Pubmed, Embase, and other relevant databases for Randomized Controlled Trials (RCTs) and observational studies evaluating zinc supplementation for olfactory dysfunction. Studies published up to April 2025 were included. Improvement in olfactory function was assessed with validated olfactory tests or subjective questionnaires. Qualitative symptoms (parosmia, phantosmia), adverse effects, patient-reported outcomes, and quality of life measured by questionnaires were evaluated.

**Results:**

A total of 6 studies, including 3 RCTs and 3 observational studies, were included in the review. Sample sizes varied between 25 and 606 participants, with follow-up durations ranging from 1 to 15 months. The meta-analysis showed no significant difference in olfactory function improvement between the zinc-treated group and the control group (OR = 1.78; 95% CI 0.63–5.03; p = 0.28). In addition, the analysis of subjective perception of improvement, also did not demonstrate a statistically significant effect (OR = 0.69; 95% CI 0.25–1.93; p = 0.48). However, an individual study reported improvement in specific subgroups, such as those with posttraumatic anosmia. Adverse events were generally mild and primarily gastrointestinal.

**Conclusion:**

Current evidence does not support a clear benefit of zinc supplementation for olfactory disorders. Further high-quality RCTs are necessary to determine its potential role, particularly in targeted subgroups.

**Level of evidence:**

Level 2a.

## Highlights

• Zinc did not significantly improve olfactory function.
• Studies showed high heterogeneity in design and outcomes.
• Level 2a evidence: zinc not routinely recommended.

## Introduction

Olfactory dysfunction, encompassing both reduced sense of smell (hyposmia) and complete loss of smell (anosmia), affects a significant portion of the global population. This condition not only diminishes the ability to detect odors but also impacts taste and overall quality of life.^1^ Common causes of olfactory disorders include upper respiratory infections, head trauma, sinonasal disease, and idiopathic conditions.^1^ In recent years, the COVID-19 pandemic has led to an increased prevalence of olfactory dysfunction due to post-viral damage to olfactory structures.

Zinc is an essential trace element involved in many physiological processes, including immune function, cell growth, and sensory functions.^2^ Its role in olfaction is linked to the regeneration of olfactory epithelium and the modulation of neural pathways related to smell.^3^ Zinc deficiency has been associated with impaired olfactory function, leading to the hypothesis that zinc supplementation might improve olfactory disorders.^4^ This systematic review aims to evaluate the efficacy and safety of zinc supplementation for the treatment of olfactory disorders.

## Methods

This systematic review and meta-analysis was conducted in accordance with the PRISMA guidelines and was retrospectively registered in the PROSPERO database (Registration nº CRD420261321935). This systematic review and meta-analysis included Randomized Controlled Trials (RCTs) and observational studies, such as cohort and retrospective cohort designs, that evaluated the effect of zinc supplementation on olfactory dysfunction. The study population consisted of adults and children diagnosed with olfactory disorders of any etiology, including post-infectious, post-traumatic, and chemotherapy-induced cases. Interventions involved any form or dosage of zinc, including zinc sulfate, zinc gluconate, or zinc oxide, compared to placebo or no treatment. The primary outcome was improvement in olfactory function, assessed through validated tests such as the University of Pennsylvania Smell Identification Test (UPSIT), Sniffin’ Sticks, Phenyl Ethyl Alcohol (PEA) odor detection threshold test or subjective improvement in olfactory perception. Secondary outcomes included reduction in parosmia and phantosmia, and incidence of adverse events.

### Search strategy

A comprehensive literature search was conducted using PubMed, Cochrane Library, Scopus, and Web of Science from database inception to April 2025. The search strategy included both Medical Subject Headings (MeSH) and free-text terms such as “zinc”, “zinc sulfate”, “zinc gluconate”, “olfactory dysfunction”, “anosmia”, and “hyposmia”. We used the following search terms in electronic databases: (“zinc” OR “zinc sulfate” OR “zinc gluconate”) AND (“olfactory dysfunction” OR “anosmia” OR “hyposmia” OR “smell impairment” OR “olfactory disorder”). Grey literature was screened using ClinicalTrials.gov and OpenGrey. References of relevant studies were also manually reviewed. No language or publication restrictions were applied. The search yielded 731 articles. After removal of duplicates and screening of titles and abstracts, ten full-text articles were reviewed. Of these, four were excluded due to absence of a control group or zinc supplementation. The remaining six studies were included in the meta-analysis.

### Data extraction and assessment of the risk of bias

Study selection was independently performed by two reviewers (MAF and HBF) based on eligibility criteria. Disagreements were resolved through consensus. Data extraction was conducted in duplicate using a standardized form to record study design, sample size, zinc dosage, follow-up duration, patient demographics, outcome measures, and results. The risk of bias was evaluated using the revised Cochrane Risk of Bias 2 (RoB2) tool for randomized controlled trials.^5^ For nonrandomized studies, the Risk of Bias in Nonrandomized Studies of Interventions (ROBINS-I) tool was applied.^6^ Any disagreements regarding the assessments were addressed through discussion and resolved by consensus between the reviewers.

### Statistical analysis

Statistical analysis was conducted using Review Manager (RevMan) version 5.4 (Cochrane, London, UK). For dichotomous outcomes, Odds Ratios (ORs) with 95% Confidence Intervals (95% CIs) were calculated. Heterogeneity was evaluated using the *I*^2^ statistic. A random-effects model was applied when *I*^2^ exceeded 50%. We used R version 4.3.2 (Foundation for Statistical Computing, Vienna, Austria) and the extension packages “meta,” “metafor”, “dmetar”, “ggplot2”, and “forestplot” for all calculations and graphics.^7^, ^8^, ^9^ Subgroup analyses were conducted according to the etiology of olfactory loss. Sensitivity analyses were performed to examine the impact of excluding studies with a high risk of bias.

## Results

From the 731 identified records, 10 full-text articles were assessed for eligibility. Six studies met the inclusion criteria and were analyzed, comprising three RCTs and three observational cohort studies.^10^, ^11^, ^12^, ^13^, ^14^, ^15^ Fig. 1 illustrates the search strategy we employed. The included studies investigated various zinc formulations and dosages, with follow-up durations ranging from 1 to 15 months. The total sample size was 1460 participants, including 436 who received zinc and 1024 controls. A detailed description of the included studies is presented in Table 1. The results of the quality assessment are presented in Table 2.

**Fig. 1:**
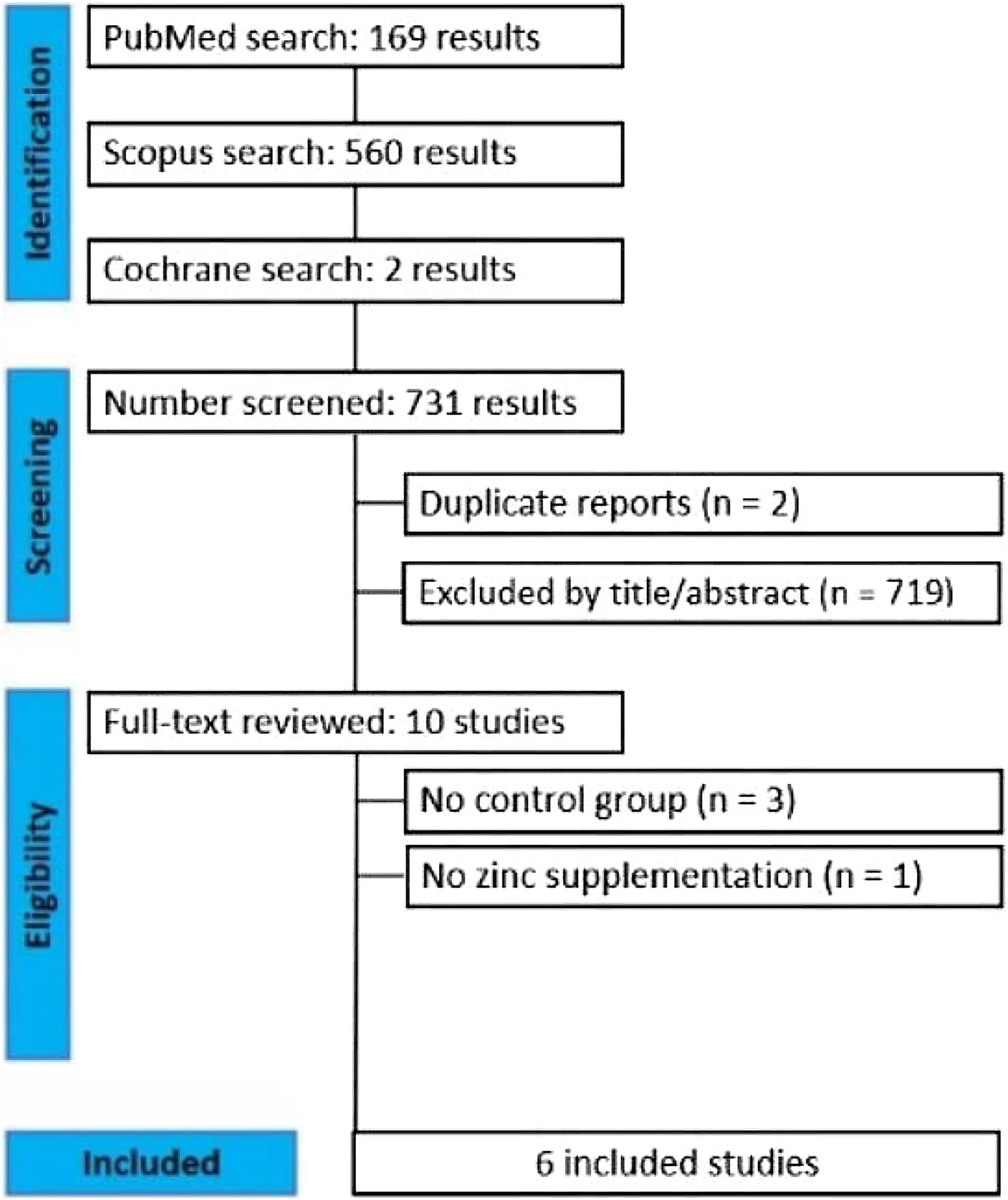
Preferred Reporting Items for Systematic Reviews and Meta-Analyses (PRISMA) flow diagram illustrating the process of study screening and selection.

**Table 1:** Baseline characteristics of included studies.

Study	Study design	Zinc dosage	Follow-up, months	Nº of patients and etiology of olfactory dysfunction	Nº of patients receiving zinc	Nº of patients not receiving zinc	Age (years), mean (range)	Male, n (%)	Olfactory tests	Improvement against control in olfactory test, OR (95% CI)	Improvement against control in subjective evaluation, OR (95% CI)	Improvement against control in parosmia and phantosmia, OR (95% CI)
Henkin RI et al., 1976	Double-blind RCT w/ placebo	Zinc sulphate, 100 mg of zinc ion in four oral doses	6	51 All etiologies	25	26	54.8 (19–84)	25 (50)	Detection and recognition thresholds	0.92 (0.31–2.77)	No difference reported; data unavailable.	No difference reported; data unavailable.
Aiba T et al., 1998	Observational Retrospective	Zinc sulphate, 300 mg/day	1	426 Postinfectious (184), posttraumatic (95) and unknown (147)	167	259	NR	NR	NA	NA	1.31 (0.89−1.94)	NR
Lyckholm L et al., 2012	Double-blind RCT	Zinc sulphate, 220 mg/day	3	58 Chemotherapy patients with smell alterations	29	29	53	17 (29.3)	NA	NA	No difference between groups	NR
Jiang R et al., 2015	RCT	Zinc gluconate 30 mg/day	3‒15	145 Posttraumatic	74	71	39.1 (15–84)	78 (53.8)	PEA	4.9 (1.7–13.9)	NR	NR
Garcia ECD et al., 2024	Observational Retrospective Cohort	Zinc oxide 17 mg/day	3	66 Postinfectious	33	33	42.7 (14–75)	32 (49)	UPSIT®	1.19 (0.38–3.8)	1.00 (0.37–2.73)	0.45 (0.1–1.98)
Fornazieri MA et al, 2024	Observational Retrospective Cohort	Zinc (different doses)	2‒3.5	714 Postinfectious	108	606	36.7 (16–71)	178 (24.9)	NA	NA	0.26 (0.25‒0.46)	NA

RCT, Randomized Controlled Trial; w, With; NR, Not Reported; NA, Not Available; PEA, Phenyl Ethyl Alcohol odor detection threshold test; UPSIT, University of Pennsylvania Smell Identification Test.

**Table 2:** Risk of bias assessment for included studies. Summary of the methodological quality of the six included studies using the Cochrane RoB2 tool for randomized controlled trials and ROBINS-I for observational studies.

Study	Design	Bias due to confounding/ Randomization	Selection of participants	Classification of interventions	Deviations from intended interventions	Missing data	Measurement of outcomes	Overall risk of bias
Henkin RI et al., 1976	RCT	Low	Low	Some concerns	Low	Low	Low	Some concerns
Lyckholm L et al., 2012	RCT	Low	Low	Low	Low	Low	Low	Low
Jiang Ret al., 2015	RCT	Low	Some concerns	Low	Low	Low	Low	Some concerns
Aiba T et al., 1998	Observational	Moderate	Low	Low	Moderate	Low	Low	Moderate
Garcia ECD et al., 2024	Observational	Moderate	Low	Low	Moderate	Low	Low	Moderate
Fornazieri MA et al., 2024	Observational	Moderate	Low	Low	Moderate	Low	Low	Moderate

The risk of bias was evaluated using the revised Cochrane Risk of Bias 2 (ROB 2) tool for randomized controlled trials and the Risk of Bias in Nonrandomized Studies of Interventions (ROBINS-I) tool for observational studies.

Regarding the primary outcomes, the pooled analysis showed no statistically significant improvement in olfactory test results among zinc-treated patients compared to controls (OR = 1.78; 95% CI 0.63–5.03; p = 0.28; *I*^2^ = 63.2%, Fig. 2). One study (Jiang et al., 2015) demonstrated a significant benefit for post-traumatic anosmia with an OR of 4.9 (95% CI 1.7–13.9), but this was not consistent across other studies. Subjective improvement in olfaction was also not significantly different between zinc and control groups. The pooled OR was 0.69 (95% CI 0.25–1.93; p = 0.48; *I*^2^ = 90.9%, Fig. 3). A single study evaluated the effect on parosmia and phantosmia, reporting a non-significant OR of 0.45 (95% CI 0.1–1.98).

**Fig. 2:**
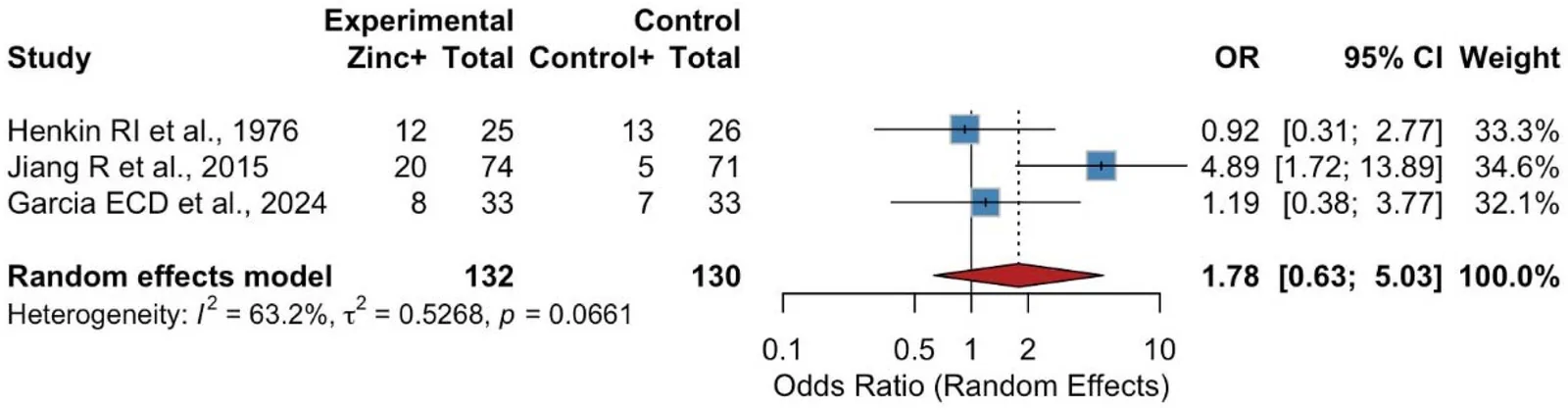
Forest Plot of Olfactory Tests Scores Improvement with Zinc Supplementation Meta-analysis of the effect of zinc on olfactory test performance (e.g., UPSIT) compared to control. Odds Ratios (ORs) and 95% Confidence Intervals (95% CIs) are shown for each study.

**Fig. 3:**
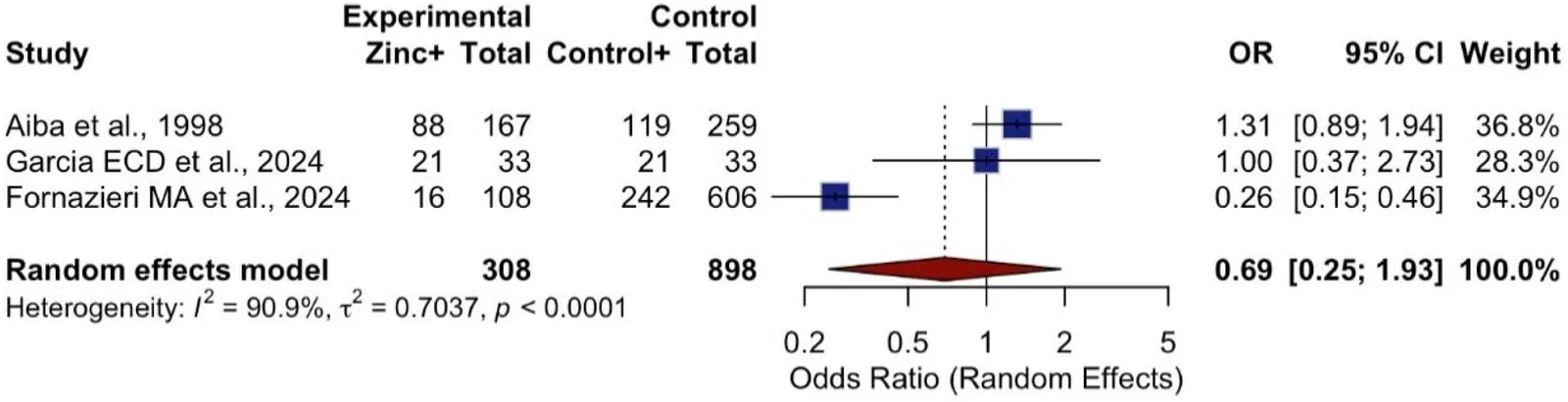
Forest Plot of Subjective Olfactory Improvement with Zinc Supplementation Meta-analysis of patient-reported olfactory improvement in zinc-treated versus control groups. Odds Ratios (ORs) and 95% Confidence Intervals (CIs) are displayed for each study.

Secondary outcomes varied across the included studies. Parosmia and phantosmia were specifically assessed only in the study by Garcia et al. (2024), which reported improvement rates associated with zinc supplementation. Patient-reported outcomes and subjective assessments of olfactory function were used in most studies, although the tools and definitions varied. Quality of life was not systematically measured in any of the included trials.

Adverse events were poorly reported overall. Only Henkin et al. (1976) explicitly described side effects, noting that they were mild and predominantly gastrointestinal in nature (e.g., abdominal cramps and diarrhea). No study reported any serious or life-threatening adverse events.

## Discussion

This comprehensive meta-analysis of six studies ‒ including three randomized controlled trials and three observational studies ‒ evaluated the efficacy of zinc supplementation in patients with olfactory dysfunction. The total sample included 1,460 participants with post-infectious, post-traumatic, and idiopathic etiologies. Zinc supplementation was not associated with a significant improvement in olfactory function based on validated olfactory tests such as UPSIT. The pooled analysis showed no statistically significant difference between the zinc and control groups (OR = 1.78; 95% CI 0.63–5.03; p = 0.28).

When subjective perception was analyzed (reported in three of the six studies), no consistent improvement was observed. The forest plot (Fig. 3) revealed that zinc did not significantly outperform no zinc supplementation in self-reported olfactory improvement. Both olfactory tests and subjective assessments consistently showed no significant benefit from zinc supplementation. This alignment reinforces the robustness of the findings, suggesting that the lack of treatment effect is not limited to a specific type of outcome measure. Although subjective tools are inherently more variable and susceptible to bias, the consistency between self-reported outcomes and psychophysical testing in this analysis supports the conclusion that zinc supplementation does not lead to meaningful clinical improvement in olfactory function.

Although no serious adverse events were reported in the included trials, safety data were inconsistently documented, with only one study (Henkin et al., 1976) explicitly describing mild gastrointestinal effects. This lack of systematic reporting limits our ability to draw firm conclusions about the tolerability of zinc supplementation, particularly at higher doses or with long-term use. Given zinc's over-the-counter availability and its perceived safety, future studies should prioritize structured adverse event monitoring to better inform clinical decision-making.

While the proposed mechanisms of zinc ‒ such as promoting neuronal regeneration and modulating inflammation in the olfactory epithelium ‒ are biologically plausible, current clinical evidence remains inconclusive.^16^ Despite the same route of administration (oral), the heterogeneity observed across studies in terms of zinc formulation (sulfate, gluconate, or oxide), dosage (ranging from 17 mg to 300 mg daily), and duration of treatment (from one to fifteen months) likely contributes to the variability in outcomes. This inconsistency highlights the need for standardized protocols in future trials, as differences in bioavailability and pharmacokinetics may substantially influence both efficacy and safety. Without such standardization, it remains challenging to determine whether the absence of benefit reflects a true lack of effect or merely methodological noise.

This analysis has several limitations. The total number of patients was modest, and most studies had limited follow-up. The types of olfactory dysfunction varied, and all etiology-specific results could not be pooled due to insufficient subgroup data. Adverse event monitoring and quality of life reporting were either lacking or not standardized across studies. The small number of included trials limited the ability to assess publication bias and precluded meta-regression for age, duration of anosmia, or baseline olfactory scores.

Despite its limitations, this meta-analysis is the first to integrate both psychophysical olfactory testing and patient-reported outcomes to evaluate the efficacy of zinc supplementation in olfactory dysfunction. A rigorous risk-of-bias evaluation was performed using RoB2 for randomized controlled trials and ROBINS-I for observational studies (Fig. 2), providing transparency and methodological robustness. Although the small number of included studies precluded extensive sensitivity or subgroup analyses, the consistency of results across diverse populations, dosing regimens, and study designs supports the overall reliability of the findings. Nevertheless, the limited sample size, heterogeneity in zinc formulations and dosages, and lack of standardized outcome measures highlight the need for further high-quality trials to clarify zinc’s clinical role.

## Conclusion

In this systematic review and meta-analysis of six studies, zinc supplementation did not demonstrate a consistent improvement in olfactory function compared to no treatment. Given the limited number of available studies and the heterogeneity across designs and outcome measures, the current evidence does not show a consistent clinical benefit of zinc for olfactory dysfunction. Larger, high-quality trials with standardized outcome measures and rigorous adverse event reporting are needed to better define zinc’s potential role in olfactory rehabilitation.

## ORCID ID

Heloísa Bortholazzi Fornazieri: 0000-0001-6544-4946

Nayara Anitelli Artero: 0000-0002-6791-6449

Ellen Cristine Duarte Garcia: 0000-0002-4083-8049

Fábio de Rezende Pinna: 0000-0002-2019-9833

Richard Louis Voegels: 0000-0003-0192-3387

## Authors’ contributions

Marco Aurélio Fornazieri, Heloísa Bortholazzi Fornazieri conducted the data collection, conducted the analyses, drafted the initial version, and revised the manuscript. Nayara Anitelli Artero, Ellen Cristine Duarte Garcia conducted the data collection, drafted the initial version, and revised the manuscript. Fábio de Rezende Pinna, Richard Louis Voegels conducted the analyses, drafted the initial version, and revised the manuscript. All authors approved the final manuscript as submitted.

## Ethical approval

This study is a meta-analysis based exclusively on data extracted from previously published articles; therefore, ethical approval was not required.

## Funding

This research did not receive any specific grant from funding agencies in the public, commercial, or not-for-profit sectors.

## Data availability statement

The data supporting the findings of this study are available upon reasonable request.

## Declaration of competing interest

The authors declare no conflicts of interest.

## References

[1] Whitcroft K.L., Altundag A., Balungwe P. (2023). Position paper on olfactory dysfunction: 2023. Rhinology..

[2] Abdelmaksoud A.A., Ghweil A.A., Hassan M.H. (2021). Olfactory disturbances as presenting manifestation among egyptian patients with COVID-19: possible role of zinc. Biol Trace Elem Res..

[3] Ishimaru T. (2022). Enhancement of olfaction by femtomolar concentrations of zinc ions. Neurosci Lett..

[4] Helman S.N., Adler J., Jafari A. (2022). Treatment strategies for postviral olfactory dysfunction: a systematic review. Allergy Asthma Proc..

[5] Sterne J.A.C., Savović J., Page M.J. (2019). RoB 2: a revised tool for assessing risk of bias in randomised trials. BMJ..

[6] Sterne J.A., Hernán M.A., Reeves B.C. (2016). ROBINS-I: a tool for assessing risk of bias in non-randomised studies of interventions. BMJ.

[7] Viechtbauer W. (2010). Conducting meta-analyses in R with the metafor package. J Stat Softw..

[8] Harrer M., Cuijpers P., Furukawa T.A., Ebert D.D. (2019). https://arxiv.org/abs//Available%20from:%20http://dmetar.protectlab.org/.

[9] Balduzzi S., Rücker G., Schwarzer G. (2019). How to perform a meta-analysis with R: a practical tutorial. Evidence Based Mental Health..

[10] Henkin R.I., Schechter P.J., Friedewald W.T., Demets D.L., Raff M. (1976). A double blind study of the effects of zinc sulfate on taste and smell dysfunction. Am J Med Sci..

[11] Aiba T., Sugiura M., Mori J. (1998). Effect of zinc sulfate on sensorineural olfactory disorder. Acta Otolaryngol Suppl..

[12] Lyckholm L., Heddinger S.P., Parker G., Coyne P.J., Ramakrishnan V., Smith T.J. (2012). A randomized, placebo-controlled trial of oral zinc for chemotherapy-related taste and smell disorders. J Pain Palliat Care Pharmacother..

[13] Jiang R.S., Twu C.W., Liang K.L. (2015). Medical treatment of traumatic anosmia. Otolaryngol Head Neck Surg..

[14] Garcia E.C.D., Rosa L.R., Santos A.C.R.D., Ishimatsu G.K.A., Lopes N.M.D., Fornazieri M.A. (2024). Investigating the efficacy of melatonin, topical sodium citrate, and multivitamin with zinc as a potential treatment for postinfectious loss of smell. Braz J Otorhinolaryngol..

[15] Fornazieri M.A., Cunha B.M., Nicácio S.P. (2024). Effect of drug therapies on self-reported chemosensory outcomes after COVID-19. World J Otorhinolaryngol Head Neck Surg..

[16] Ikushima H., Suzuki J., Hemmi T. (2023). Effects of zinc deficiency on the regeneration of olfactory epithelium in mice. Chem Senses..

